# Examining the Effect of Assimilation Overlap on Discrimination of English and Persian Stop–Fricative Contrasts in Chinese Listeners

**DOI:** 10.3390/bs16040562

**Published:** 2026-04-09

**Authors:** Youngja Nam

**Affiliations:** Centre for Creative Convergence Education, Hanyang University, 55 Hanyangdeahak-ro, Sangnok-gu, Ansan 15588, Gyeonggi-do, Republic of Korea; nature2024@hanyang.ac.kr

**Keywords:** Perceptual Assimilation Model, speech perception, assimilation overlap, overlap scores, Chinese listeners, English and Persian stop–fricative contrasts

## Abstract

Research on cross-language adult speech perception shows that non-native speech sounds are interpreted through the listener’s L1 phonological system. According to the Perceptual Assimilation Model (PAM) and its extension, PAM-L2, discriminability of non-native/L2 speech contrasts is determined by how two phones are assimilated to L1 phonological categories. Specifically, discriminability varies depending on perceived overlap with L1 phonological categories. This study assessed the PAM/PAM-L2 account of the assimilation–discrimination relationship in discrimination of non-native/L2 stop–fricative contrasts, focusing on how discrimination varies with assimilation overlap. Chinese listeners completed assimilation and AXB discrimination tasks with six English (/p-f/, /b-v/, /t-θ/, /t-s/, /d-ð/, /d-z/) and two Persian (/k-x/, /g-ɣ/) stop–fricative contrasts. The contrasts were assimilated as four Uncategorized–Categorized (UC) contrasts, one with no overlap and three with partial overlap, and four Two-Category (TC) contrasts. The discrimination results showed that TC and non-overlapping UC contrasts were more accurately discriminated than partially overlapping UC contrasts, consistent with PAM/PAM-L2. Further analysis revealed that overlap scores were strongly negatively correlated with discrimination accuracy at the group level, and this correlation was also significant for most contrasts at the individual level. These findings suggest that exploring assimilation overlap may help clarify the assimilation–discrimination relationship in non-native/L2 stop–fricative contrast discrimination.

## 1. Introduction

Research on adult cross-language speech perception has shown that the phonological system of a listener’s native language (L1) influences how they perceive non-native speech contrasts (Fabra & Tyler, 2023; Faris et al., 2018; Georgiou, 2020, 2022, 2024; Nam, 2025; Tyler et al., 2014). Listeners often have difficulty discriminating non-native contrasts that are not phonemic in their L1. However, non-native contrasts do not always pose the same level of difficulty. For example, in Nam’s (2018) study of perception of English affricate–fricative contrasts, Korean learners of English (L2) were highly accurate at discriminating English /ʧ-ʃ/ and /ʧ-s/ with both phonemes mapped onto two different Korean phonemes. Similarly, Georgiou (2020) reported that naïve Russian listeners discriminated Greek stop–fricative and fricative–fricative contrasts at rates exceeding 90% accuracy, with two consonants perceived as belonging to distinctive L1 phonemic categories.

Two widely cited frameworks in cross-language speech perception are the Speech Learning Model (SLM; Flege, 1995) and the Perceptual Assimilation Model (PAM, and its extended version for L2 learners, PAM-L2) (Best, 1995; Best & Tyler, 2007). SLM focuses on L2 speech learning in terms of how individual L2 speech sounds relate to existing L1 phonetic categories, whereas PAM/PAM-L2 focuses on how L2 speech contrasts are perceived in relation to L1 phonological categories. This study draws on PAM/PAM-L2 for theoretical underpinnings because it specifically predicts the discrimination accuracy of an L2 speech contrast based on how its members are mapped onto L1 phonological categories.

Numerous studies have examined the perception of non-native consonant contrasts within the PAM/PAM-L2 framework (e.g., Antoniou et al., 2013; Georgiou, 2020; Georgiou et al., 2020; Nam et al., 2021; Tyler, 2020). With respect to stop–fricative contrasts, only two studies (Georgiou, 2020; Georgiou et al., 2020) have addressed stop–fricative contrast perception by examining naïve Russian listeners’ perception of Greek stop–fricative contrasts. To date, no studies have investigated stop–fricative contrast perception using contrasts other than Greek or in listeners from language backgrounds other than Russian within the PAM/PAM-L2 framework. Thus, it remains underexplored whether PAM/PAM-L2 principles can account for the perception of other stop–fricative contrasts and for non-Russian L1 populations. In addition, to the author’s knowledge, no prior research has explicitly addressed Chinese listeners’ perceptual patterns for non-native or L2 stop–fricative contrasts. The present study fills these gaps by testing the PAM/PAM-L2 account of the relationship between assimilation types and discrimination performance in Mandarin Chinese (Chinese, hereafter) listeners’ perception of English (L2) and Persian (non-native) stop–fricative contrasts, focusing on how assimilation overlap accounts for discriminability of non-native/L2 stop–fricative contrasts.

### 1.1. PAM/PAM-L2

According to the PAM, naïve listeners interpret unfamiliar non-native speech sounds through their L1 phonological categories. This perceptual assimilation can result in one-to-one mappings between non-native and L1 categories (i.e., categorized) or one-to-multiple mappings (i.e., uncategorized). Categorized cases occur when a non-native phone is perceived as similar to a given L1 category. It should be noted that similarity can range from low similarity, where the non-native phone is a poor exemplar of the L1 category, to high similarity, where it is a prototypical exemplar of that category. Uncategorized cases occur when a non-native phone does not perceptually match any L1 category. A non-native phone is considered as categorized or uncategorized depending on whether a non-native-to-L1 match meets a categorization criterion, typically set at 50% or 70% (Antoniou et al., 2013; Bundgaard-Nielsen et al., 2011; Fabra & Tyler, 2023; Faris et al., 2016, 2018; Tyler et al., 2014). Based on non-native-to-L1 assimilation patterns, PAM predicts the degree of perceptual difficulty for non-native contrasts. In an extended version of PAM for L2 speech perception, PAM-L2 (Best & Tyler, 2007) incorporates L2 learners’ developing phonetic and phonological knowledge of the target language, thereby extending PAM’s predictions to L2-to-L1 assimilation patterns and allowing that the L2-to-L1 assimilation may evolve with increased experience.

The PAM/PAM-L2 posits six assimilation types of non-native phones, each predicting perceptual difficulty. In the Two-Category (TC) type, two non-native phones are assimilated into distinct L1 categories. Discrimination is expected to be excellent since each phone is perceived as belonging to a separate category. In the Single-Category (SC) type, both phones are assimilated to the same L1 category with the same degree of category goodness. Discrimination is predicted to be poor. In the Category-Goodness (CG) type, both phones are assimilated to the same L1 category, but one is perceived as a better exemplar than the other. This contrast is expected to result in moderate to good discrimination. In the Uncategorized–Categorized (UC) type, one phone is assimilated to an L1 category, while the other is not mapped to a single L1 category. Discrimination is generally expected to be very good. In the Uncategorized–Uncategorized (UU) type, neither phone is assimilated to an L1 category, and discrimination may range from poor to good depending on their phonetic similarity. Finally, in the Non-Assimilable (NA) type, both phones are perceived as non-speech sounds because they fall outside the L1 phonological system. Discrimination in this type is expected to vary.

Many studies have supported the predicted relationship between assimilation types and discrimination (Antoniou et al., 2012; Best et al., 2001; Hallé et al., 1999; Bohn & Best, 2012). Nevertheless, UC and UU contrasts have received relatively less attention. Motivated by this, Faris et al. (2016) conducted a systematic examination of uncategorized responses in their study of Australian English vowel contrasts by Egyptian Arabic listeners and proposed three types—focalized, clustered, and dispersed—in which an uncategorized L1 response category is chosen to identify a given non-native phone. In a focalized assimilation, a non-native phone is mapped to a single L1 category at an above-chance rate; in a clustered assimilation, it matches multiple L1 categories above chance; and in a dispersed assimilation, no single L1 category is consistently chosen above chance. Faris et al. (2016) proposed that uncategorized assimilations can be further differentiated into focalized, clustered, and dispersed types. In addition, they defined UC- and UU-assimilated contrasts as non-overlapping, partially overlapping, and completely overlapping, depending on how many response categories the two phones share at above-chance levels.

In a subsequent study, Faris et al. (2018) explicitly evaluated their 2016 proposal of the role of perceived phonological overlap in the perception of uncategorized assimilations by testing naïve Australian English listeners with Danish monophthong and diphthong contrasts. They found that discrimination of UC and UU vowel contrasts was generally influenced by the degree of overlap with L1 categories, thereby supporting Faris et al.’s (2016) view that discrimination of UC and UU contrasts depends on overlap with L1 categories.

### 1.2. Overlap Scores

As an attempt to provide a quantitative measure for predicting discrimination accuracy in L2 vowel perception, Flege and MacKay (2004) introduced an L2-to-L1 assimilation-based overlap score approach, showing that discrimination tended to decrease as the degree of overlap between L1 categories increased. When two L2 phones exhibit overlap in their assimilation to L1 categories, the overlap scores are calculated by summing the lower assimilation percentages from the overlapping categories (see Section 3.2). Building on this approach, Levy (2009) investigated American English listeners’ perception of French vowels across three experience groups (no, moderate, and extensive). Levy (2009) found that vowel contrasts with lower overlap were generally discriminated more accurately than those with higher overlap. For example, the /y-i/ contrast with less than 1% overlap was discriminated with only 4% error rates, whereas the /œ-o/ contrast with over 60% overlap yielded much higher error rates (39%). In addition, overlap scores tended to decrease with increased L2 experience, suggesting that assimilation patterns may change over time with increased exposure.

Further support for the predictive role of overlap scores in L2 vowel perception came from Baigorri et al. (2019). They examined both assimilation patterns and discrimination accuracy for American English vowels in early and late L1 Spanish-L2 English bilinguals. The bilinguals generally found it more difficult to discriminate English vowel contrasts with higher assimilation overlap, whereas they were more accurate at discriminating English vowel contrasts in which phones were mapped onto distinct L1 categories. Interestingly, even early bilinguals with extensive L2 exposure displayed non-native-like perception for certain contrasts, suggesting that assimilation overlap may serve as a quantitative index for explaining variability in L2 vowel perception across different levels of L2 experience.

### 1.3. The Role of Overlap in Consonant Perception

The PAM’s distinctions of overlap degree, based on non-native vowel perception (Faris et al., 2016, 2018), have also been applied to consonant perception. Georgiou (2020) examined perceptual patterns for Greek stop–fricative /t-θ/, /d-ð/, and /g-ʝ/, and fricative–fricative /ç-x/ contrasts in naïve Russian listeners (see also Georgiou et al., 2020, for another study using the same Greek stimuli with Russian listeners). Russian listeners assimilated /t-θ/ as non-overlapping UC and /d-ð/, /g-ʝ/, and /ç-x/ as non-overlapping UU and discriminated these non-overlapping contrasts well, with discrimination scores ranging from 84% to 93%, consistent with the extended PAM view (Faris et al., 2016, 2018).

Tyler (2020) directly assessed whether L2 learners, particularly those with classroom-based language training, exhibit phonological overlap between L2 phones and whether this overlap changes with immersion experience by examining Japanese listeners’ perception of the synthetic English /r-l/ continuum. Results from identification and forced category-goodness rating tasks showed that at the /l/-end, stimuli were rated equally as good instances of both /l/ and /r/. However, at the /r/-end, stimuli were rated as better instances of /r/ than /l/. More experienced listeners exhibited greater distinction in their ratings at both the /r/- and /l/-ends, but the difference was smaller than that of native English listeners. In other words, less experienced Japanese listeners showed more category overlap than their more experienced counterparts. As the author pointed out, this suggests that in PAM’s terminology, the /r-l/ contrast was perceived as overlapping assimilation by less experienced Japanese listeners. Discrimination performance also improved as phonological overlap decreased with increased immersion experience. These findings point to the influence of the degree of overlap in L2 consonant perception.

The degree of overlap with L1 categories also plays a role in the perception of non-native stop contrasts. Nam et al. (2021) reported that Korean-naïve Quebec French listeners whose native system distinguishes stops with a two-way voicing contrast yielded SC and partially overlapping UC assimilations for Korean three-way stop contrasts. The results showed that assimilation overlap generally affects the discriminability of Korean stop contrasts. For example, at the labial and coronal places, French listeners showed greater sensitivity to fortis-aspirated contrasts (e.g., /p*-p^h^/), which were assimilated as partially overlapping UC, than to lenis-aspirated contrasts (e.g., /p-p^h^/), which were assimilated as SC. This pattern aligns with the PAM view (Faris et al., 2018) in which SC, akin to completely overlapping UC or UU assimilations, is more challenging than partially overlapping UC or UU assimilations.

### 1.4. Studies of Perception of English Stops and Fricatives by Chinese Listeners

Prior work on Chinese learners’ English consonant perception has primarily targeted fricatives and affricates (e.g., Lan, 2020; Lin & Liu, 2025; Y. Zhang & Xiao, 2014). No studies have been explicitly designed to examine Chinese listeners’ perceptual patterns for English stop–fricative contrasts. However, a few studies have reported how Chinese learners of English perceive English stops and fricatives. For example, Gong and Zhou (2015) examined 24 English consonants, including both stops and fricatives, in intervocalic position with high- and low-experience Chinese listeners to test the effect of language experience on assimilation and identification. In both groups, English consonants with L1 counterparts such as stops and some fricatives like /f, s/ were consistently assimilated to a single Chinese category, whereas consonants without L1 equivalents (e.g., /θ, ð/) showed more dispersed assimilation across several Chinese categories. In addition, the high-experience group exhibited more concentrated assimilation patterns (e.g., English /z/ was mapped onto Chinese /ts/) than the low-experience group (e.g., English /z/ was mapped onto both Chinese /ts/ and /s/). L2-to-L2 identification results indicated that consonants with dispersed assimilations were generally less accurately identified as intended than those with more concentrated assimilations. These patterns suggest that L2-to-L1 assimilation can be influenced by L2 experience.

De Jong et al. (2025) investigated how listeners of Taiwanese Mandarin perceive English labial and coronal stops (/p, b, t, d/) and non-sibilant fricatives (/f, v, θ, ð/) using four prosodic locations to explore how L2-to-L1 mapping patterns account for L2 identification performance. They compared the identification results with the accuracy levels predicted from the mapping data. In consonant–vowel syllables, in PAM’s terminology, English stops exhibited one-to-one L2-L1 assimilations, whereas English fricatives (/v, θ, ð/) displayed one-to-many assimilations, except for /f/ with one-to-one assimilation, similar to the pattern reported by Gong and Zhou (2015). Specifically, English stops were identified at levels consistent with mapping-based predictions, indicating strong influence from L1 categories, whereas English fricatives exceeded the mapping-based predictions, suggesting that listeners’ fricative perception was not primarily determined by L1 categories but reflected categorization shaped by L2 experience. The results further revealed that manner distinctions (stop vs. fricative) were better predicted from the mapping data than voicing distinctions (voiceless vs. voiced). Overall, these findings suggest that cross-language mapping remains strongly tied to L1 categories, while language experience also contributes to enhanced L2 identification beyond the predictions based on L1 categories.

### 1.5. The Present Study

As mentioned earlier, limited data (Georgiou, 2020; Georgiou et al., 2020) are available regarding stop–fricative contrast perception with the PAM/PAM-L2 framework. The present study primarily aimed to examine how perceptual assimilation types relate to discrimination performance in the perception of English and Persian stop–fricative contrasts. Specifically, discrimination data were analyzed in relation to the degree of assimilation overlap. When UC or UU assimilations emerged, the principles of PAM/PAM-L2 (Faris et al., 2016, 2018) were tested, hypothesizing that discrimination of non-native stop–fricative contrasts assimilated to distinct L1 categories would be more accurate than contrasts involving assimilation overlap. The assignment of UC and UU assimilations was further classified according to the degree of overlap. Specifically, when no overlap occurred, the contrast was assigned UC-N or UU-N; when partial overlap occurred, the contrast was assigned UC-P or UU-P; and when complete overlap occurred, the contrast was assigned as UC-C or UU-C (Fabra & Tyler, 2023; Faris et al., 2018; Nam, 2025). The secondary focus was to examine how discrimination accuracy varies as a function of overlap scores to further elucidate the influence of assimilation overlap in discrimination performance. Overlap scores were computed at both group and individual levels, and this study is the first to investigate consonant contrast perception using overlap scores at both levels.

An assimilation task and an AXB discrimination task were conducted. In the assimilation task, Chinese listeners were asked to map English stops /p, b, t/ and fricatives /f, v, θ, s, ð, z/ and Persian stops /k, g/ and fricatives /x, ɣ/ onto their L1 consonant categories. In the AXB task, Chinese listeners discriminated six English stop–fricative contrasts (/p-f/, /b-v/, /t-θ/, /t-s/, /d-ð/, and /d-z/) and two Persian velar stop–fricative contrasts (/k-x/ and /g-ɣ/). The Persian stimuli were included to provide velar stop–fricative contrasts since English lacks stop–fricative contrasts at the velar place, thereby covering all three places of articulation. In Chinese, stops consist of /p, p^h^, t, t^h^, k, k^h^/ and fricatives of /f, s, ɕ, ʂ, ʐ, x/ (X. Yu & Do, 2024; Zhao & Li, 2009). Thus, all test contrasts are non-phonemic in Chinese, except for /p-f/, /t-s/, and /k-x/.

## 2. Materials and Methods

### 2.1. Participants

Thirty-five native speakers of Chinese aged 21–34 (mean age = 25.9 years; SD = 3.1; 30 females, 5 males) participated. All were international students enrolled at universities in Seoul, Korea, and their length of residence in Korea ranged from 1 month to 5.3 years, with a mean of 2.1 years (SD = 1.5). At the time of the study, 28 participants had intermediate Korean proficiency, and 7 participants had beginning level based on Test of Proficiency in Korean (TOPIK) scores. Self-reports indicated that all participants had basic to lower-intermediate English proficiency, except for two who reported upper-intermediate proficiency. None was fluent in any other foreign language or had lived in any other foreign country prior to their relocation to Korea for their studies. None reported a history of hearing or language disorders. All participants were compensated for their participation.

### 2.2. Stimuli

The stimuli consisted of ten English consonants /p, b, f, v, d, ð, z, t, θ, s/ and four Persian consonants /k, x, g, ɣ/ produced in a /CVC/ context in which all stimuli shared the same vowel and final consonant /as/ but differed in their initial consonants (i.e., pas, bas, fas, vas, das, ðas, zas, tas, θas, sas, kas, xas, gas, ɣas). All test syllables were nonwords in both English and Persian. Four native speakers of North American English (2 males and 2 females) and four native Persian speakers (2 males and 2 females) were recorded in a sound-attenuated booth at the Infant Speech Perception Lab at McGill University. All English and Persian speakers were in their 20s and were graduate students at McGill University, except for one speaker. The Persian speakers were born and educated in Iran before relocating to Canada for their studies, with a length of residence in Canada ranging from 1 to 2 years. The stimuli were recorded in the carrier phrase, “I will say CVC again”, in English and “Man dobare CVC az aval mikhaham beguyam” in Persian, using a Sennheiser microphone. Recordings were made using the Praat software (Version 5.1. 44, Boersma & Weenink, 2010) and an LG X Note E300 laptop with a sampling rate of 44,100 Hz. Each speaker produced each token multiple times, and two tokens per syllable were selected based on an intelligibility test by three independent English-speaking and three Persian-speaking judges. The final stimulus set included 80 English tokens, in which two tokens were selected from each of the ten test syllables for each of the four speakers, and 32 Persian tokens, in which two tokens were selected from each of the four test syllables for each of the four speakers. All English and Persian speakers and judges were compensated for their participation.

### 2.3. Procedure

Participants performed the two perceptual tasks individually in a quiet place. The Paradigm Player software (Tagliaferri et al., 2010) was used to present the stimuli and collect responses. The stimuli were delivered to the participants through headphones. All instructions shown on the computer screen were provided in Chinese. No feedback was provided during the tests.

In the AXB discrimination task, listeners were presented with a triad of test syllables. AXB trials included four trial types (AAB, ABB, BAA, and BBA) in which two initial consonants were the same and one differed (e.g., /pas/-/pas/-/fas/). Listeners were instructed to click “first” if the middle initial consonant was the same as the first one or “third” if it was the same as the third one. The AXB task consisted of a brief practice block, followed by eight test blocks, each containing 64 trials. Each block included two trials for each of the four AXB trial types and for each of the eight contrasts (/pas-fas/, /bas-vas/, /tas-θas/, /tas-sas/, /das-ðas/, /das-zas/, /kas-xas/, and /gas-ɣas/), which resulted in 512 trials (16 trials for each AXB type for each contrast). In each AXB trial, the tokens were produced by a pair of male and female speakers, and each member of the same phoneme categories was represented by different tokens (e.g., p_1_-f_1_-f_2_). Tokens were counterbalanced across AXB trials and within each trial type for each contrast and speaker. The order of the block presentation and trial types within each block were randomized for each participant, and the inter-stimulus interval was set to 1 s. Participants could take self-paced breaks lasting up to 5 min after every two blocks of 128 trials.

In the assimilation task, participants selected the Chinese consonant label that best matched the initial consonant of each stimulus (b, p, m, f, d, t, n, l, g, k, h, j, q, x, zh, ch, sh, r, z, c, s, y, w) (X. Yu & Do, 2024; Y. Yu et al., 2019; F. Zhang & Yin, 2009). Then, participants rated how well the English and Persian consonants matched the Chinese category they selected, using a 5-point scale (1 = very dissimilar, 5 = very similar). After a brief practice block, participants completed eight test blocks, each including two tokens for each of the 14 consonants (p, f, b, v, t, θ, s, d, ð, z, k, x, g, ɣ). This totaled 112 tokens per participant. Blocks and tokens within each block were randomly presented for each participant.

## 3. Results

### 3.1. Assimilation Results

Table 1 presents the assimilation patterns of the English and Persian stimuli by Chinese listeners. Percentages of English and Persian consonants chosen as the most similar Chinese categories were averaged across listeners, and the mean category-goodness ratings are shown in parentheses. By applying a categorization criterion of 70% (Fabra & Tyler, 2023; Nam et al., 2021; Nam, 2025, Tyler et al., 2014), a test consonant was considered categorized if it was identified as a single Chinese category in 70% or more of listeners’ responses; otherwise, it was classified as uncategorized. When a test consonant was uncategorized, one-sample *t*-tests were conducted to examine how many Chinese categories were chosen at rates significantly above chance (4.35%) to identify the consonant. Based on this analysis, the given consonant was labeled as focalized when it showed a single above-chance mapping, clustered when it showed multiple above-chance mappings, or dispersed when it showed no above-chance mappings (Faris et al., 2016, 2018; Fenwick et al., 2017; Nam et al., 2021; Nam, 2025).

Chinese listeners consistently identified English /p/ and /f/ as Chinese /p^h^/ (91.4%) and /f/ (87.5%), perceived as good exemplars of those categories (4.1 and 4.0 out of 5), forming TC assimilation for /pas-fas/. For English /bas-vas/, Chinese listeners categorized /b/ as Chinese /p/ (87.5%) and /v/ as /w/ (76.1%), with ratings of 3.9 and 3.4, respectively, yielding TC assimilation. Chinese listeners almost exclusively identified English /t/ as Chinese /t^h^/ (98%) with a rating of 4.0, whereas they did not categorize English /θ/, with responses spread across Chinese /f/ (50%) and /s/ (36.8%), which received ratings of 3.9 and 3.6, respectively. Thus, /tas-θas/ formed UC assimilation. They also categorized English /s/ as Chinese /s/, with a rating of 3.0, resulting in TC assimilation for English /tas-sas/. Chinese listeners categorized English /d/ (92.1%) as a good version of Chinese /t/ (4.0), whereas they did not meet the 70% criterion for English /ð/, with responses spread across /ts/ (38.2%), /t/ (19.3%), /ɻ/ (11.8%), and /w/ (10.4%). Thus, /das-ðas/ yielded UC assimilation. They categorized English /z/ as Chinese /z/ (78.6%) with a rating of 3.6, forming TC assimilation for /das-zas/. Chinese listeners categorized Persian /k/ (90.7%) as Chinese /k^h^/ with a rating of 4.0, but did not categorize Persian /x/, with 65.7% of responses mapped onto Chinese /k^h^/ and 24.3% to /x/. Thus, /kas-xas/ was assimilated as UC. They reliably identified Persian /g/ as Chinese /k/ (97.1%) with a rating of 3.9, but their responses for Persian /ɣ/ were spread across Chinese /k/ (63.9%), /x/ (14.6%), and /k^h^/ (9.3%), yielding UC assimilation for /gas-ɣas/.

As mentioned above, a further analysis was performed with the uncategorized consonants /θ, ð, x, ɣ/ to determine whether they are assimilated as focalized, clustered, and dispersed. As shown in Table 1, these consonants exhibited clustered assimilations, with two or more Chinese response categories (indicated in boldfaced italics) selected above chance. For English /θ/, the above-chance Chinese response categories were /f/ (*t*(34) = 9.05, *p* < 0.0001) and /s/ (*t*(34) = 6.64, *p* < 0.0001), forming a UC-N assimilation for English /tas-θas/. For English /ð/, the above-chance Chinese response categories were /t/ (*t*(34) = 3.07, *p* = 0.002), /ts/ (*t*(34) = 5.45, *p* < 0.0001), /ɻ/ (*t*(34) = 1.88, *p* = 0.038), and /w/ (*t*(34) = 2.28, *p* = 0.015), yielding a UC-P assimilation with overlap on /t/ for English /das-ðas/. For Persian /x/, the above-chance Chinese response categories were /k^h^/ (*t*(34) = 11.20, *p* < 0.0001) and /x/ (*t*(34) = 3.60, *p* < 0.001), resulting in a UC-P assimilation with overlap on /k^h^/ for Persian /kas-xas/. For Persian /ɣ/, the above-chance Chinese response categories were /k/ (*t*(34) = 11.70, *p* < 0.0001), /x/ (*t*(34) = 2.56, *p* = 0.008), and /k^h^/ (*t*(34) = 2.31, *p* = 0.013), yielding a UC-P assimilation with overlap on /k/ for Persian /kas-ɣas/.

Based on the assimilation data, discrimination performance for the English and Persian stop–fricative contrasts was predicted from the extended PAM perspective (Faris et al., 2016, 2018). TC and UC-N contrasts were expected to be easily discriminated, while UC-P contrasts were expected to be less accurately discriminated. Accordingly, the expected discrimination pattern is: (TC /pas-fas/ = TC /bas-vas/ = TC /tas-sas/ = TC /das-zas/ = UC-N /tas-θas/) > (UC-P /das-ðas/ = UC-P /kas-xas/ = UC-P /gas-ɣas/).

### 3.2. AXB Discrimination Results

**Figure 1:**
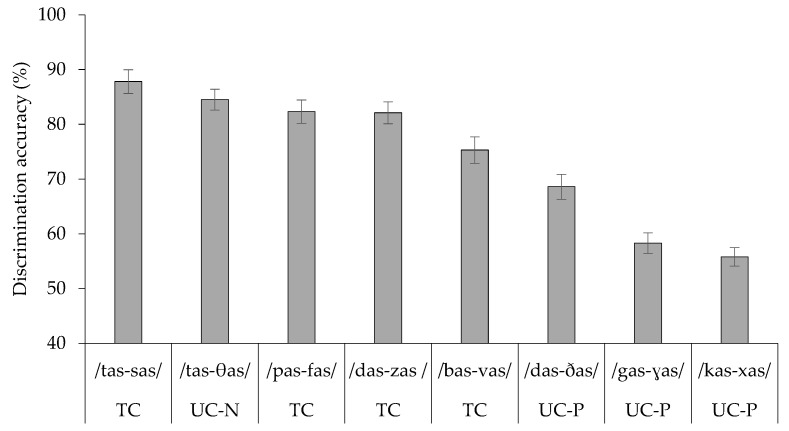
Mean discrimination accuracy for each of the eight stop–fricative contrasts, with assimilation types indicated. Error bars represent the standard error.

Percent correct discrimination scores were computed for each listener. The data were analyzed using a linear mixed-effects model, with contrast as a fixed effect and random intercepts for subjects (SPSS, version 25). There was a significant effect of contrast (*F*(7, 238) = 80.31, *p* < 0.001), showing that discrimination accuracy significantly differed across contrasts. Figure 1 illustrates the mean percent correct performance for each contrast.

Bonferroni pairwise comparisons showed that TC /tas-sas/ did not differ significantly from UC-N /tas-θas/ (diff. = 3.11, *p* = 1.0, CI [−2.98, 9.20]), TC /pas-fas/ (diff. = 5.37, *p* = 0.16, CI [−0.72, 11.46]), or TC /das-zas/ (diff. = 5.43, *p* = 0.15, CI [−0.66, 11.52]). These four contrasts were all discriminated more accurately than TC /bas-vas/: TC /tas-sas/ (diff. = 12.40, *p* < 0.001, CI [6.31, 18.49]), UC-N /tas-θas/ (diff. = 9.30, *p* < 0.001, CI [3.20, 15.38]), TC /pas-fas/ (diff. = 7.00, *p* = 0.009, CI [0.94, 13.12]), and TC /das-zas/ (diff. = 7.00, *p* = 0.01, CI [0.88, 13.06]). TC /bas-vas/ was discriminated more accurately than UC-P /das-ðas/ (diff. = 6.70, *p* = 0.017, CI [0.62, 12.80]), UC-P /gas-ɣas/ (diff. = 17.10, *p* < 0.001, CI [10.97, 23.15]), and UC-P /kas-xas/ (diff. = 19.40, *p* < 0.001, CI [13.34, 25.52]). UC-P /das-ðas/ was discriminated more accurately than UC-P /gas-ɣas/ (diff. = 10.30, *p* < 0.001, CI [4.25, 16.43]), and UC-P /kas-xas/ (diff. = 12.70, *p* < 0.001, CI [6.62, 18.80]). There was no significant difference between UC-P /gas-ɣas/ and UC-P /kas-xas/ (diff. = 2.37, *p* = 1.0, CI [–3.72, 8.46]). Accordingly, the discrimination pattern is summarized as: (TC /tas-sas/ = UC-N /tas-θas/ = TC /pas-fas/ = TC /das-zas/) > TC /bas-vas/ > UC-P /das-ðas/ > (UC-P /gas-ɣas/ = UC-P /kas-xas/).

As shown in Table 2, group-level overlap scores were computed to further explore the relationship between overlap scores and discrimination accuracy (e.g., Fabra & Tyler, 2023; Faris et al., 2018; Levy, 2009; Nam et al., 2021). The scores were obtained by summing the smaller percentages for the shared Chinese categories for each contrast across listeners (Flege & MacKay, 2004; Levy, 2009). For example, for the English contrast /das-ðas/, both phones were mapped onto the same Chinese categories (/f/, /t/, /s/, /ts/, and /k/) (see Table 1). The smaller percentages for these categories are 0.7%, 19.3%, 0.4%, 4.2%, and 2.1%, and their sum (27.1%) represents the overlap score for this contrast reported in Table 2. A Spearman rank correlation analysis assessed the relationship between discrimination accuracy and overlap scores at both the group and individual levels. At the group level, a highly significant negative association was observed (*ρ* = −1.0, *p* < 0.001), indicating that Chinese listeners performed more poorly as overlap scores increased. Pearson’s correlation likewise confirmed a very strong negative association (*r* = −0.98, *p* < 0.001).

Table 3 presents individual-level overlap scores, along with SDs and overlap ranges, to illustrate listener variability; these values were then used to assess correlations with discrimination accuracy (Fabra & Tyler, 2023; Levy, 2009). The individual-level overlap scores were computed for each contrast for each listener. These analyses revealed significant negative associations for TC /pas-fas/ (*ρ* = −0.533, *p* = 0.001), TC /bas-vas/ (*ρ* = −0.398, *p* = 0.018), UC-N /tas-θas/ (*ρ* = −0.314, *p* = 0.027), UC-P /das-ðas/ (*ρ* = −0.419, *p* = 0.001), and UC-P /kas-xas/ (*ρ* = −0.278, *p* = 0.030). By contrast, no significant associations were found for TC /das-zas/ (*ρ* = 0.131, *p* = 0.360) or UC-P /gas-ɣas/ (*ρ* = 0.116, *p* = 0.367). For TC /tas-sas/, the group-level overlap score was 1.1% (see Table 2), but at the individual level, all listeners showed 0% overlap, making correlation analysis impossible.

## 4. Discussion

The primary goal of this study was to assess the general PAM/PAM-L2 principles, which posit that discriminability of non-native/L2 stop–fricative contrasts can be predicted based on how listeners interpret the contrasts relative to their L1 phonological categories. The principles were specifically tested with respect to the role of assimilation overlap by comparing the discriminability of non-overlapping assimilation types with that of overlapping assimilation types. The second aim was to examine how discrimination accuracy varies in relation to overlap scores by analyzing the correlation between discrimination accuracy and overlap scores at both the group and individual levels. These aims were tested through assimilation and AXB discrimination tasks.

It is important to note that this study has a sample limitation because all Chinese listeners were residing in Korea, with most having intermediate Korean proficiency, which raises the possibility that their perceptual patterns have been influenced by their L3 Korean-language experience. This is particularly discussed in relation to Chinese listeners’ perceptual assimilation patterns.

### 4.1. Perceptual Assimilation Patterns

The English stops /p, t, b, d/ and the Persian stops /k, g/ were consistently categorized as Chinese stops /p^h^, t^h^, p, t/ and /k^h^, g/, respectively. The English fricatives /f, s/, which have Chinese equivalents, were also categorized as intended. However, most of the English and Persian fricatives that are not part of the Chinese inventory were uncategorized, assimilating across multiple Chinese categories. Among these, English /ð/ showed the widest response spread, with above-chance mappings to five Chinese categories (/ts, t, ʂ, w, l/). Interestingly, the Persian /x/ was also uncategorized and more frequently mapped onto Chinese /k^h^/ than Chinese /x/. This might be attributed to the fact that the Chinese velar fricative /x/ shares acoustic similarities with the glottal fricative /h/ (Lee et al., 2012), which may lead Persian /x/ to be perceptually more similar to Chinese /k^h^/ than to Chinese /x/. By comparison, English fricatives /v/ and /z/, which lack Chinese equivalents, were consistently mapped onto a single Chinese phonological category.

The English-to-Chinese assimilation patterns observed in this study are consistent with those reported by Gong and Zhou (2015). There were two exceptions: /v/ and /z/. In the present study, English /v/ was categorized as Chinese /w/, while Gong and Zhou (2015) found that English /v/ was uncategorized in both high- and low-experience groups. In the present study, English /z/ was categorized as Chinese /ts/, whereas in Gong and Zhou’s (2015) study, this categorization was only observed in the high-experience group. It should be noted that the most frequent L1 response categories for the uncategorized English /v/ and /z/ were /w/ and /ts/, respectively, in Gong and Zhou’s (2015) study, which employed VCV syllables. In this regard, the present study provides further empirical evidence of how Chinese listeners interpret English stops and fricatives in relation to their L1 categories, irrespective of syllabic position.

To further explore perceptual patterns for English stops and fricatives in Chinese listeners who were in a Korean-immersive environment, it is useful to consider how Korean listeners perceive English consonants relative to Korean phonological categories. Schmidt (1996) examined assimilation patterns for 22 English consonants in Korean listeners whose language has only three fricatives: /s/, /s/, and /h/. In the context of /Ca/ syllables, Korean listeners categorized the English stops /p, t, b, d/ as their respective Korean stops /p^h^, t^h^, p, t/. English fricatives /v/ and /z/ were categorized as Korean /p/ and /ʧ/, respectively, while other English fricatives were uncategorized. English /f/ was primarily assimilated to Korean /p^h^/, while English /ð/ and /θ/ were mainly assimilated across Korean /t/ and /t*/ and across /s*/ and /t*/, respectively. English /s/ was assimilated across Korean /s/ and /s*/. These patterns suggest that Korean listeners generally perceived English fricatives that are absent in the Korean phonetic inventory as Korean stops, while also possibly reflecting how L1 Korean may shape the perception of English stops and fricatives. However, this does not rule out the possibility that L3 Korean might have influenced the perception of these consonants in Chinese listeners in this study.

### 4.2. Discrimination Accuracy

The AXB discrimination results revealed that, as expected, Chinese listeners were more accurate at discriminating TC and non-overlapping UC assimilations than partially overlapping UC assimilations. This performance pattern supports the PAM/PAM-L2 principles in which discrimination of TC assimilation is excellent, while discrimination of UC assimilation is comparable to TC but becomes challenging when two phones have overlap with L1 phonological categories. Specifically, the observed hierarchy within UC assimilations (UC-N > UC-P) aligns with the extended PAM/PAM-L2 view (Faris et al., 2016, 2018), in which overlap with L1 categories modulates the discriminability of UC assimilations, while suggesting that assimilation overlap affects the discriminability of non-native/L2 stop–fricative contrasts.

The second aim of this study was to examine discrimination performance relative to overlap scores by computing overlap scores for each contrast at both the group and individual levels (Fabra & Tyler, 2023; Levy, 2009). This approach was designed to gain a deeper understanding of how the degree of assimilation overlap influences discrimination performance. A closer inspection of the discrimination data revealed that not all UC-P contrasts were equally discriminated. Specifically, the UC-P contrast /das-ðas/ was more accurately discriminated than the other UC-P contrasts /gas-ɣas/ and /kas-xas/, which partially deviates from the PAM/PAM-L2 predictions. For /das-ðas/, /d/ (categorized as Chinese /t/) showed primary overlap with the second most frequent L1 response category for /ð/, while overlap with the first most frequent L1 response category was small (4.6%). In contrast, both /gas-ɣas/ and /kas-xas/ approached within-category assimilations, in which both phones were mapped onto the same L1 category in 63.9% of responses.

These patterns suggest that the small overlap (4.6%) between the dominant L1 response categories for /das-ðas/ may have led to more accurate discrimination than the within-category-like contrasts /gas-ɣas/ and /kas-xas/. This finding aligns with the observation that listeners showed better discrimination for an L2 vowel contrast with no overlap between dominant L1 categories than those with overlap between them (Nam, 2025). Furthermore, these patterns also point toward the role of overlap since /das-ðas/ exhibited an overlap score of 27.1%, while /gas-ɣas/ and /kas-xas/ had overlap scores of 66.4% and 71.4%, respectively. These findings also suggest broad implications for instructional approaches, particularly in identifying target contrasts for perceptual training. For example, English contrasts /bas-vas/ and /das-ðas/ and Persian contrasts /kas-xas/ and /gas-ɣas/ were less accurately discriminated than the remaining contrasts and posed perceptual challenges for Chinese listeners. These contrasts could serve as focal points in perceptual training.

Additionally, a hierarchy in discrimination was observed for TC contrasts, which is also partially inconsistent with PAM/PAM-L2 predictions. Chinese listeners performed similarly well on the TC contrasts /tas-sas/, /pas-fas/, and /das-zas/, but their discrimination of the TC contrast /bas-vas/ was significantly poorer than the other TC contrasts. This hierarchy in discrimination performance can be explained by the overlap scores (see Table 2). The overlap scores for /tas-sas/ (3.6%), /pas-fas/ (3.9%), and /das-zas/ (6.8%) were small relative to /bas-vas/ (16.1%). These differences in assimilation overlap suggest that the degree of assimilation overlap plays a role in discrimination accuracy. Furthermore, an examination of the fricative members of TC contrasts indicated that the category-goodness rating for /v/ (3.4) was low relative to /f/, /s/, and /z/ (ranging from 3.6 to 4.0). This suggests that /v/ was likely perceived as a less stable exemplar of Chinese /w/, and this perceptual uncertainty may have hindered the discrimination of /bas-vas/.

Although SLM (Flege, 1995) is not the primary focus of this study, it provides some insight into the observed discrepancy. Specifically, for /das-ðas/, /d/ was perceived as an existing L1 category, while /ð/ was perceived as distinct from any L1 phonetic category. Within SLM, /ð/ forms a separate phonetic category for /ð/, which facilitates its discrimination from /d/. In contrast, SLM’s concept of equivalence classification applies to the within-category-like contrasts /gas-ɣas/ and /kas-xas/, in which both phones are likely perceived as variants of the same L1 category, leading to poorer discrimination.

Overall, these findings suggest that assimilation patterns and assimilation overlap may modulate discrimination of non-native or L2 stop–fricative contrasts not only across different assimilation types but also within the same assimilation types. The observed discrepancies in discrimination within the same assimilations extend beyond the predictions of the PAM/PAM-L2 framework and indicate that overlap scores provide a quantitative measure that helps explain discrimination patterns, particularly in cases where PAM’s predictions do not align with the data. Within PAM/PAM-L2, contrasts classified as the same assimilation type are expected to yield similar levels of discrimination, which makes it challenging to account for the discrepancies in discrimination within the same type of TC or UC-P assimilation. PAM/PAM-L2 also raises a problem for accounting for the lower discrimination accuracy of TC /bas-vas/, which exhibited a noticeably larger overlap score compared to the other TC contrasts. These cases exploit the quantitative overlap score approach as a supplementary tool, offering a measurable dimension that complements the qualitative aspect of PAM/PAM-L2.

Importantly, these perceptual patterns suggest that integrating both PAM/PAM-L2 and overlap scores can offer a more comprehensive understanding of discrimination of non-native/L2 stop–fricative contrasts. This approach will be particularly useful in capturing the role of assimilation overlap occurring between non-dominant L1 response categories, such as in the case of /bas-vas/. Additionally, the case of UC-P contrasts suggests that such an integrative approach can provide further insight into these discrepancies in discrimination of non-native/L2 stop–fricative contrasts by examining whether substantial overlap occurs between two dominant L1 response categories. These findings reflect the gradient nature of speech perception, suggesting that such an integrative account can shed light on the nuances of cross-language speech perception.

### 4.3. Correlation Between Overlap Scores and Discrimination Accuracy at the Individual Level

In the analysis of the correlation between overlap scores and discrimination accuracy, a strong negative correlation was observed at the group level, suggesting that as overlap scores for the English and Persian stop–fricative contrasts increased, their discriminability decreased (see Table 2), similar to vowel studies (Fabra & Tyler, 2023; Flege & MacKay, 2004; Levy, 2009; Nam, 2025). The analysis was then extended to the individual level. At the individual level, significant negative correlations were observed for /pas-fas/, /bas-vas/, /tas-θas/, /das-ðas/, and /kas-xas/. However, /tas-sas/, /das-zas/, and /gas-ɣas/ did not show significant correlations. Similarly, in the vowel perception study, Fabra and Tyler (2023) also reported that discrimination accuracy was negatively correlated with overlap scores at the group level, while the correlation was only observed for some vowel contrasts at the individual level.

Upon examining the individual data, as shown in Table 3, no overlap was observed for /tas-sas/, which likely resulted in a null correlation. Although /das-zas/ (4.6%) showed a slightly higher overlap score than /pas-fas/ (2.1%) and /tas-θas/ (3.2%), a significant correlation was found for /pas-fas/ and /tas-θas/ but not for /das-zas/. A closer inspection of the individual data revealed that only three listeners exhibited overlap for /das-zas/, whereas four and six listeners exhibited overlap for /pas-fas/ and /tas-θas/, respectively. The lack of a significant correlation for /das-zas/ may be partially attributable to the small number of listeners showing overlap. Additionally, /das-zas/ exhibited twice the variability compared to /pas-fas/ and /tas-θas/. When variability was calculated across only the listeners who showed overlap, /das-zas/ (SD = 38.2%) displayed considerably greater variation than /pas-fas/ (SD = 7.2%) and /tas-θas/ (SD = 10.5%), which may have further obscured the correlation. It should also be noted that for /bas-vas/ and /das-ðas/, overlap was observed in 14 and 20 listeners, respectively, with SDs of 29.7% and 28.5%.

Both UC-P contrasts /kas-xas/ (67.9%) and /gas-ɣas/ (65.4%) exhibited high overlap scores with similar variability, with overlap observed in 33 and all 35 listeners, respectively. Despite the high overlap, only /kas-xas/ showed a significant negative correlation with discrimination accuracy. Given that these contrasts involved within-category-like cases such as SC or CG contrasts, a further analysis was performed with their category-goodness ratings. The results showed that /gas-ɣas/ (3.9 vs. 3.2, Cohen’s d = 0.73) exhibited a larger difference than /kas-xas/ (4.0 vs. 3.7, Cohen’s d = 0.28). This perceptual disparity reflected in rating differences may potentially have contributed to the lack of correlation for /gas-ɣas/.

Additionally, these results point to the potential for individualized perceptual training, where each L2 learner’s training approach is tailored based on their contrast-specific overlap scores and discrimination patterns. For example, an individual listener’s overlap score-discrimination patterns for target L2 contrasts could be useful in instructional settings for systematically tracking whether the two phones of those contrasts are perceptually distinct.

## 5. Conclusions

This study is generally consistent with the PAM/PAM-L2 principles by observing that non-native and L2 stop–fricative contrasts are more accurately discriminated when assimilated to distinctive L1 phonological categories than when they overlap with L1 categories. Overlap scores accounted for a hierarchy of discrimination not only between different assimilation types but also within the same assimilation types, with discriminability decreasing as a function of overlap scores, offering further insight into the degree of assimilation overlap in predicting discrimination of non-native/L2 stop–fricative contrasts. Additionally, this study suggests that analyzing the relationship between assimilation overlap and discrimination performance at the individual level may provide a more nuanced understanding of listener variability in the perception of non-native/L2 stop–fricative contrasts.

It is important to note that this study had instances of only non-overlapping and partially overlapping UC assimilations, with no completely overlapping instances, which limits the scope for fully testing the PAM/PAM-L2 predictions regarding the assimilation–discrimination relationship. Future research should examine a broader range of assimilation types, specifically UC or UU assimilations with varying degrees of overlap, to further explore the effect of assimilation overlap on shaping discrimination of non-native or L2 stop–fricative contrasts. One meaningful direction would be to investigate listeners from various L1 backgrounds, particularly those whose L1 inventories contain fewer fricatives than Chinese, which could yield a wide variety of UC or UU assimilations. As pointed out earlier, another limitation is that all Chinese listeners had beginning to intermediate Korean proficiency. It remains unclear whether the results reflect the influence of L1 Chinese in the perception of English and Persian stop–fricative contrasts or whether they could be influenced by L3 Korean experience. Thus, the findings should be interpreted with caution. Further research is needed to determine whether similar perceptual patterns would be observed in Chinese listeners residing in China. This also suggests an important direction for future research to explore how Korean listeners perceive English and Persian stop–fricative contrasts. Additionally, the lack of correlation between overlap scores and discrimination for some contrasts at the individual level suggests that more data are needed to further explore how overlap scores relate to discrimination in non-native/L2 stop–fricative contrasts.

## Figures and Tables

**Table 1:** Mean percent assimilation of English and Persian stop and fricative consonants in terms of Chinese consonant categories. “C” and “U” represent “Categorized” and “Uncategorized,” respectively. Mean category-goodness ratings are shown in parentheses, ranging from 1 (very dissimilar) to 5 (very similar).

		English and Persian Stimuli													
		pas	bas	fas	vas	θas	ðas	tas	das	sas	zas	kas	gas	xas	ɣas
		C	C	C	C	U	C	C	C	C	C	C	C	U	U
Chinese response categories	p^h^	**91.4** ^a^ **(4.1)**	2.1	0.4				0.4			0.4		0.4	1.8	2.5
	p	1.1	**87.5 (3.9)**	1.4	9.3	1.4	0.4			0.4	0.4	0.4			
	f	0.4	0.4	**87.5 (4.0)**	2.1	***50.0*** ^b^ ***(3.9)***	1.1	0.4	0.7	1.1	0.4				0.7
	t^h^	2.5		0.7		2.5	0.7	**98.2 (4.0)**			0.7 (2.0)	1.4		2.9	
	t		3.9	0.4	0.4	1.1	***19.3 (3.5)***		**92.1 (4.0)**	0.7	0.4	0.4	1.1		0.7
	s			3.6		***36.8 (3.6)***	1.1		0.4	**86.8 (3.8)**	3.9	0.7		0.4	
	ts	0.4	1.1	2.1	4.6	3.2	***38.2 (3.3)***	0.4	4.6	5.0	**78.6 (3.6)**	0.4	0.7	0.7	1.1
	k^h^	2.9	0.4	0.4	0.4		0.4	0.4				**90.7 (4.0)**		***65.7 (3.7)***	***9.3 (3.3)***
	k	0.7	2.5	1.4	2.5	0.4	3.2		2.1	0.4	1.1	2.1	**97.1 (3.9)**	0.4	***63.9 (3.2)***
	x	0.4	0.4		0.4								0.4	***24.3 (3.6)***	***14.6 (2.8)***
	ts^h^			0.7		0.4	0.4	0.4		0.7		3.2	0.4	3.2	0.4
	tʂ					0.7	3.9				3.6			0.4	
	ʂ					2.1				5.0	0.4				
	tɕ^h^					0.4									0.4
	tɕ						0.4				1.1				
	ɻ	0.4	0.4		4.3		***11.8 (2.9)***				8.9	0.4			4.6
	l			0.4			8.9					0.4			0.7
	w		1.4	1.1	**76.1 (3.4)**	1.1	***10.4 (3.3)***				0.4			0.4	1.1

Numbers indicate the percentages of the English and Persian initial consonants assimilated to Chinese consonant categories. ^a^ Boldfaced non-italicized numbers indicate that the English and Persian initial consonants reached the 70% categorization criterion. ^b^ Boldfaced italicized numbers indicate that the uncategorized Chinese response categories were chosen at above-chance levels. Chinese response labels obtaining 0% are not included. Category-goodness ratings are only provided for the above-chance response categories.

**Table 2:** Group-level overlap scores and mean discrimination accuracy for each stop–fricative contrast.

PAM Type	Contrast	Overlap Score (%)	Discrimination Accuracy (%)
TC	/tas-sas/	1.1	87.8
UC-N	/tas-θas/	3.6	84.5
TC	/pas-fas/	3.9	82.3
TC	/das-zas/	6.8	82.1
TC	/bas-vas/	16.1	75.3
UC-P	/das-ðas/	27.1	68.6
UC-P	/gas-ɣas/	66.4	58.3
UC-P	/kas-xas/	71.4	55.8

**Table 3:** Individual-level overlap scores for each stop–fricative contrast.

PAM Type	Contrast	Overlap Score (%)	SD	Overlap Range (%)
TC	/tas-sas/	0	0	0
UC-N	/tas-θas/	3.2	8.2	0–37.5
TC	/pas-fas/	2.1	6.4	0–25
TC	/das-zas/	4.6	18	0–87.5
TC	/bas-vas/	12.9	24.3	0–100
UC-P	/das-ðas/	24.3	30.2	0–87.5
UC-P	/gas-ɣas/	65.4	29.6	13–100
UC-P	/kas-xas/	67.9	30.8	0–100

## Data Availability

The original contributions presented in this study are included in the article. Further inquiries can be directed to the corresponding author.
